# Proposed New Electrolytic Conductivity Primary Standards for KCl Solutions

**DOI:** 10.6028/jres.096.008

**Published:** 1991

**Authors:** Y. C. Wu, W. F. Koch, K. W. Pratt

**Affiliations:** National Institute of Standards and Technology, Gaithersburg, MD 20899

**Keywords:** cell constant, conductance, demal, electrolytic conductivity, molal, potassium chloride, primary standards, resistance, specific conductance

## Abstract

An absolute determination of aqueous electrolytic conductivity has been made for 0.01 molal (*m*) and 0.1 *m* potassium cliloride solutions, over the temperature range of 0 to 50 °C in 5 degree intervals. A cell with a removable center section of accurately known length and area was used for the measurements. Values were adjusted to be in conformity with the ITS-90 temperature scale. The overall uncertainty over the entire temperature range is estimated to be 0.03%. Values at 25 °C for 0.01 and 0.1 *m* are 0.00140823 and 0.0128246 S/cm, respectively. It is proposed that these values be adopted as primary standards for aqueous electrolytic conductivity, replacing the demal scale.

## 1. Introduction

Parker and Parker [1] introduced the unit of “demal” to denote the concentration scale for electrolytic conductivity standards some 60 years ago. Since then the electrolytic conductivities (specific conductances) for that unit have been subjected to two major revisions. One was by Jones and Bradshaw [2] about 10 years later, and the other was based on recalculations [3,4,5] due to changes in basic constants and measurement scales, such as the international ohm to absolute ohm, and the temperature scale from the International Practical Temperature Scale, IPTS-48, to IPTS-68. Now a new International Temperature Scale (ITS-90) has been adopted as of January 1, 1990 [6]. Hence, the old values for the conductivity standards have to be revised again.

In 1987, we reviewed the primary and secondary conductivity standards [3] and stated that “although these changes have affected the values of standards to 0.1% or less, this is significantly greater than the claimed accuracy of the original measurements. Moreover, the ‘demal’ unit is not a customary unit of concentration in solution chemistry, and 50 years is a long time for any given standard to go without remeasurement and verification.” For these reasons, we redetermined the primary standards in 1989, based on a conductance cell with a well-defined geometry whose dimensions were accurately measured, and concluded that … future adoption of the molality scale as the basis for the primary standards for specific conductance would be desirable when data for other molalities and temperatures are available [7].

We have recently completed measurements of the conductivities of two aqueous solutions of KCl, based on the molal (*m*) scale, having broad application, viz., 0.01 and 0.1 *m.* Measurements were made from 0 to 50 °C at 5 °C intervals. For a better documentation of this determination we shall redescribe the cell construction, the apparatus, and the procedures of measurements [7,8].

## 2. The Design of the Conductance Cell

### 2.1 The Principle

The electrolytic conductivity (specific conductance), κ, of a given material is the reciprocal of ρ, the resistivity. By definition

R=ρ(l/A) (1)

where *R* is the resistance, *l* is the length and *A* is the cross sectional area of the given material. Thus, if *l/A* is already known for a measured *R*, ρ and 1/κ are determined. Since the cell constant in a Jones-type conductance cell is determined by the lines of force between the two electrodes, the center section of the tube can be removed in order to shorten the distance between the electrodes, thereby reducing the resistance. Subsequently, the same section can be put back, lengthening the distance and increasing the resistance, provided that the lines of force are not disturbed. The difference in resistance is due to the geometry of the displacement of the center tube and the resistivity (or electrolytic conductivity) of the solution in question. If the geometry of the tube, *l/A*, and the difference in resistance are known, the electrolytic conductivity can be determined, independent of any reference material; ergo, it is an absolute determination. This principle can be expressed as follows:

$$R_{N}=\rho G_{N}$$ (2)

$$R_{w}=\rho \left(G_{N}+l/A\right)=\rho G_{w}$$ (3)

$$R_{w}-R_{N}=\rho l/A$$ (4)

$$1/\rho \equiv \kappa =l/A\left(R_{w}-R_{N}\right)$$ (5)

where *G*_N_ and *G*_w_ are the cell constants of the cell without and with the center tube, respectively; *l/A* is the length-to-cross sectional area of the center tube, called the cell constant, *G*_T_; and *R*_N_ and *R*_w_ are the measured resistances for the cell without and with the center tube, respectively. It should be noted that κ, the electrolytic conductivity of the given solution, includes the contribution of the solvent.

### 2.2 The Construction of the Cell

In order to insert the center tube into the cell, the cell must be cut into two halves, which can be connected together with or without the center tube. When they are connected together, the inside diameter of the joint or joints must be streamlined and there must be no leakage.

**Figure 1:**
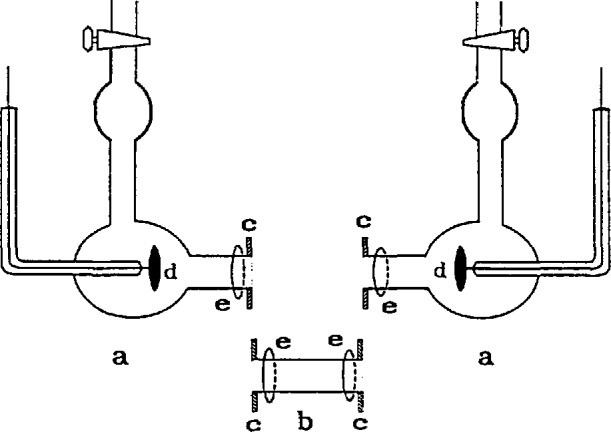
Component parts of the cell, a, half cells; b, center tube; c, flanges; d, platinum electrodes; e, o-rings.

To meet these requirements, a length of precision-bore 1-cm I.D. Pyrex^1^ tubing, with uniformity certified by the manufacturer, was cut into three sections. A prefabricated flange with an I.D. of 1.3 cm, O.D. of 2.5 cm and thickness of 0.65 cm was epoxied onto each of the tubes such that the face of the flange was flush with the cut end of the tube. Then, each flanged end was ground to optical flatness. The unflanged end of each of the singly flanged tubes was joined to the corresponding electrode chamber, each of which contained a 2 cm (diameter) platinum disk electrode. The electrodes were gold soldered to a 2 mm platinum wire that extended through a graded-glass seal. The component parts of the cell are shown schematically in figure 1.

**Figure 2:**
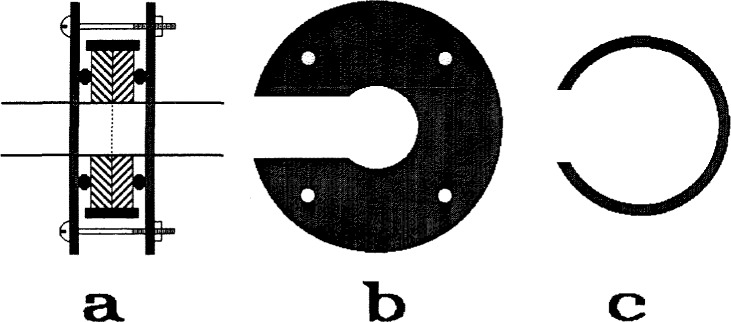
Detail of the flange assembly, a, overall cross section; b, side view of holder; c, side view of c-ring.

**Figure 3:**
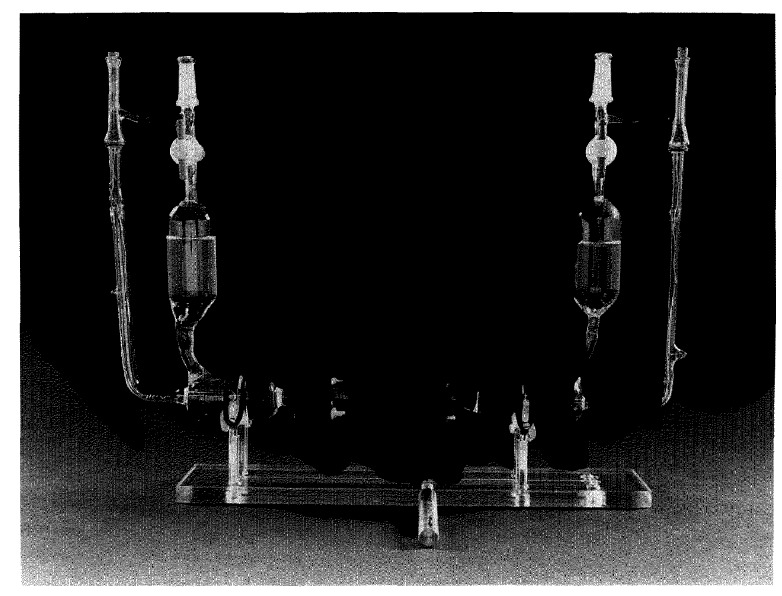
Photograph of cell assembly with holder.

To assemble the cell, the mating flange ends were held together by a C-shaped Bakelite band which could be slightly enlarged with a little pressure, such that the two flanges fit snugly into it. In this way the flanges were prevented from moving laterally. Lateral and rotational movements were prevented by a Bakelite two-plate assembly tightened together with four nylon screws and nuts. A rubber O-ring was inserted between each plate and the rear side of the flange. This assembly is shown schematically in figure 2. The whole cell assembly with holder is shown in figure 3.

### 2.3 The Determination of the Cell Constant

From eq (5), the electrolytic conductivity is determined from the cell constant, *G*_T_ ≡ *l/A*, of the center tube. For the cell used in this work, the length *l* and the I.D. of the center tube were determined by the Length and Mass Division of the National Institute of Standards and Technology. The reported mean values are as follows:

$$\begin{array}{l} l=\;8.00046\pm 0.00019\;\text{cm},\\ D=1.00634\pm 0.00005\;\text{cm},\\ A=0.79539\pm 0.00008\;{\text{cm}}^{2}. \end{array}$$

Thus

$$G_{T}=l/A=10.0585\pm 0.0013\;{\text{cm}}^{-1}.$$

The relative value of the uncertainty in *l* indicates that the two flanges on the tube are not exactly parallel; the axes of the tubes are off by a maximum of 0.02 degree, which is established by the limit of accuracy of the instruments used to fabricate the cell.

### 2.4 Temperature Effect on Cell Constant

The cell constant is defined as the effective length between the two electrodes over the effective area of the electrodes, *l/A*. In a Jones-type of cell, the effective *l/A* is controlled by the size of the center tube, according to eq (5). The conductance is exactly determined by the removable section of the center tube, i.e., *G*_T_. The temperature effect on *G*_T_ can be expressed [9] as

$$\begin{array}{c} \frac{1}{G_{T}}\frac{dG_{T}}{dt}\approx \frac{1}{l}\frac{dl}{dt}-\frac{1}{A}\frac{dA}{dt}\\ =\alpha_{g}-2\alpha_{g}=-\alpha_{g} \end{array}$$ (6)

where α_g_ = 3.6 × 10^−6^ °C^−1^, the thermal expansion coefficient of Pyrex glass. For platinum, α_pt_ = 9 × 10^−6^ °C^−1^. Over the whole range of experimental temperatures, i.e., 0 to 50 °C, Δ*G*_T_ is approximately 0.02%. However, *G*_w_ and *G*_N_ are not changed in the same way as in eq (6), for there is no means to determine the cell dimension of the entire cell shown in figure 3. The only way to determine the temperature effect on *G*_w_ and *G*_N_ is through eqs (2) and (3), at a series of temperatures.

### 2.5 Consistency

At a given temperature, each of the three cell constants, *G*_w_, *G*_N_, and *G*_T_ (of which only two are independent), has a fixed value which is independent of the conductivity of a solution. Thus, by eliminating κ from eqs (2) and (3), we obtain for any two solutions A and B at a given temperature *t:*

$${\left(R_{w}/R_{N}\right)}_{A}=G_{w}/G_{N}={\left(R_{w}/R_{N}\right)}_{B}$$ (7)

where subscripts A and B solutions A or B. This consistency was verified for *t* = 25 °C in the previous report [7]. The same principle has been applied for each temperature in this present study.

## 3. Experimental

### 3.1 Apparatus

Three major instruments are required for these measurements: the cell, a constant temperature bath for the cell, and an ac bridge with a null detector.

The cell has been described in the preceding section. The constant-temperature bath is a rectangular, steel, open-top box surrounded by a wood cabinet with a hinged cover. The space between the five sides of the steel box and the wood cabinet, about 8 cm, is insulated with glass wool. Within the steel box, there are vertical inner walls situated about 5 cm from the sides and front of the steel box and 15 cm from the rear side, which end about 10 cm from both the top and the bottom of the box. A length of 0.635 cm (0.25 in) diameter copper tubing is looped twice around the outside of the inner walls, and leads to the outside of the wood cabinet for connection to a temperature-controlled fluid circulating system. An 8 cm diameter, 25 cm long trough is located at the bottom of the steel box and is centered on and close to a stirrer, located at the rear bottom of the steel box. This stirrer forces the bath fluid out through the trough and over the rim of the inner walls, resulting in good circulation. On the front side of the steel box, two studs are mounted to provide a support for the cell. The support is adjustable and allows the cell to be manipulated to a desirable position.

The fluid previously used for the constant temperature bath was light-weight petroleum oil [7]. It performed poorly because at low temperature its viscosity increased; air-bubbles produced by the Stirrer were entrained, which caused nonuniform thermal conductivity. Temperature control degraded to ± 0.02 °C. To improve the viscosity and to avoid trapped air, a 50-50 mixture of liglit-weight petroleum oil and “Petroleum spirits” was used. Additional styrofoam insulation was attached to the bath enclosure. With the new fluid and insulation, the bath temperature was controlled to ±0.005 at 0°C, ±0.001° at 25 °C, and ±0.002° at 50 °C. An ice bath was also tried for 0 °C. However, oil was drawn into the cell through the joints, due to the sudden drop in temperature (from 25 to 0 °C). Thus, all the results reported were obtained through the use of the modified constant temperature oil bath. A small circulating cryogenic bath, whose temperature was controlled to ±0.05 °C, was used to cool the oil bath.

A cooling fluid, a mixture of ethylene glycol and water, at a constant temperature of 1.5 °C below that of the experiment, was circulated by pumping through the copper coil and back into the control chamber. The heating of the oil bath was facilitated with a 250 W quartz heater (Corning) submerged into the bath. In the heater circuit, a 60 W light bulb was connected in series so that the power of the heater was reduced. The whole heating unit was connected to a proportional temperature control unit that had been modified to decrease the maximum temperature bandwidth from 0.1 to 0.03 °C. A thermistor was used as the sensor for the temperature controller. The quartz thermometer and frequency counter used as the bath temperature indicator had been calibrated with the NIST standard thermometer to the accuracy of 1 mK. The quartz thermometer was calibrated every 3 months at the beginning. After 1-1/2 years, the calibration drift rate gradually diminished. After 3 years, the calibration was stable to within 1 to 2 mK. The room temperature of the laboratory was controlled at 23.5 ± 0.5 °C.

**Figure 4:**
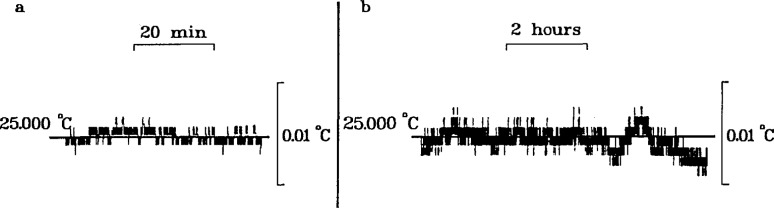
Temperature control of the oil bath, a, short-term (1 h) control with adjustment; b, long-term (8 h) control without adjustment.

When the temperature controlling equipment was correctly adjusted, the temperature deviation rarely exceeded ± 0.005 °C over an extended period, e.g., overnight. However, the temperature could readily be controlled to ±0.001 °C during experiments with minor adjustments of the heating rate. This is demonstrated in figure 4.

The bridge was a Jones bridge equipped with capacitance compensation specifically designed for conductance measurements [10,11]. This bridge also employed a Wagner ground to minimize the effects of stray capacitance on the accuracy of the null point. Because the bridge was relatively old, the bridge resistors were recalibrated by the Electricity Division at NIST before beginning this experiment. The overall accuracy of the bridge was within ±0.005% without correction and ±0.001% with correction, using the recalibrated values. The lead resistance for the connections from the binding posts of the bridge to the electrodes of the cell was determined separately and was 0.301 Ω. For measurements of the cell resistance, the observed values read from the bridge were corrected both for the lead resistance and for the recalibration of the bridge resistors.

Alternating current was supplied to the bridge from a signal generator. The generator was modified by floating the secondary of the output transformer to obtain an ungrounded output, which was required to make use of the Wagner ground of the Jones bridge. A single-ended (unbalanced) output was obtained from a secondary winding of the output transformer and was used as the phase reference for the detector. The applied ac voltage to the bridge was 1.2 V RMS. A differential input preamplifier, tuned amplifier and oscilloscope were used in the detector circuit. The differential input of the preamplifier maintained the balance of the Wagner ground and eliminated the need for an input transformer. The tuned amplifier was used in the band-pass mode at a *Q* of 10 to 50, as required, to attenuate sufficiently the second and higher harmonics at the null point. The output of the tuned amplifier and the reference signal from the signal generator were connected to the vertical and horizontal inputs of the oscilloscope, respectively. The displayed pattern on the oscilloscope was a Lissajous figure and was used to indicate simultaneously both capacitive and resistive balance of the bridge [12]. The technique was superior to using the in-phase and quadrature output meters of the lock-in amplifier to indicate resistive and capacitive balance of the bridge.

With this setup, a 0.0010% change in cell resistance was readily detected on the oscilloscope. Hence, a millidegree change in the bath temperature (0.002% in *R*) was easily observed, and the uncertainty in the null point measurement did not contribute significantly to the overall uncertainty of the measurement.

The frequency generator could be operated from 20 Hz to 15 kHz. The normal operating range of frequency co was 1 to 5 kHz. The polarization effect could be corrected by extrapolating *R* versus to ω^−1^ to ω =∞ [9].

The frequency counter for the quartz thermometer was connected to a digital-to-analog converter, the output of which was monitored using a strip chart recorder. This setup was capable of monitoring small changes in temperature (100 millidegree full-scale). Because of the thermal conductivity of the oil (bath fluid) and the path between the thermometer and the conductivity cell, there was a time lag of 2 min for the two corresponding temperatures, and a minimum of 10 min at constant temperature was required to ensure that the resistance reading is at the indicated temperature.

### 3.2 Material

It has been stated previously that “KCl solutions were adopted as standard because of the stability of these solutions and the ease with which KCl could be purified” [3]. The KCl used was NIST Standard Reference Material (SRM) 999. The purity of this SRM was certified at 99.99_1_% based on K, and at 99.98_1_% based on Cl. The fine crystalline KCl was ignited at 500 °C for 4 h and stored in a desiccator before use. A batch of SRM 999 KCl was recrystallized twice and ignited at 500 °C. No noticeable difference in conductance was observed between the original SRM 999 and the recrystallized material. The SRM 999 was thus used throughout, without recrystallization.

The in-house distilled water was passed through a deionizing column before use. When the water was freshly deionized, the electrolytic conductivity, κ, was 0.2 μS cm^−1^; after it was stored in a polyethylene bottle for a few days, κ was equal to 1±0.1 μS cm^−1^, and was stable at 25 °C. Because of the CO_2_ content in the atmosphere, it was preferable to let the solution attain equilibrium with the CO_2_, rather than avoiding the CO_2_.

At other temperatures, the CO_2_ content was, of course, different than at 25 °C. The CO_2_ equilibrium was established for each temperature after thermal cycling. The electrolytic conductivities of the water at various temperatures were determined with a cell design similar to the one used by Daggett, Blair, and Kraus [13], which had a cell constant of 0.055302 cm^−1^ at 25 °C. The deviation at any given temperature was about ± 1%, which was less than 0.02 μS/cm, far beyond the uncertainty of experimental results for the electrolytic conductivities of the standard KCl solutions.

Two kg each of 0.01 and $0.1\;m\;\left(\text{mol}/{\text{kg}}_{H_{2}O}\right)$ KCl solution were prepared. All solution weights were corrected to vacuum. The solutions were stored in polyethylene bottles for a few days to reach equilibrium with atmospheric CO_2_ before use.

### 3.3 Procedure

When the cell was first made, it was cleaned with chromic acid cleaning solution, washed with water, soaked in a water bath overnight, and then vacuum dried at room temperature. If the water washing, soaking and vacuum drying did not remove the trace of adsorbed chromate, the process was repeated. Although the use of chromic acid cleaning solution is not recommended, other strong acids and organic solvents were tried without satisfaction. After the cell was clean and dry, it was assembled and filled with 0.01 *m* KCl solution. If no leak was detected at the flanged joints after the filled cell stood for an hour, the cell was put into the bath. It normally took about 30 to 40 min for the cell to reach thermal equilibrium after being put into the bath. The reading was then recorded. If no drift was noted for another hour, the bath was adjusted to the next temperature and the process was repeated three times. If the deviation of the results was random within ±0.005%, the mean was taken as the final result. If the deviation showed a trend toward increasing or decreasing resistance, there were two probable causes: cell contamination or loss of water from the solution by evaporation. Cell contamination was generally caused by adsorption of foreign substance(s) on the walls and electrodes. There was no easy way out of this except to repeat the experiment until the foreign substances were leached out and the readings were constant. If the drift was due to concentration increase by evaporation, a fresh solution had to be used and the experiment repeated. (The evaporation occurred while-transferring solution from the bottle to the cell, a process that was performed in the open. Since the rate of evaporation was about 3 mg per min and the transferring processes took about 2 min, a total of about 6 mg of water was lost, approximately 0.003% of the total volume. Because of this, no bottle of solution was used twice.)

The procedures for changing from one concentration to another, or for replacing the center tube in the cell, were similar. Caution was always exercised to prevent the oil on the outside of the cell from creeping inside. If this occured, the cleaning process was repeated.

The bath temperature was generally started at 25 °C, and was either raised or lowered in 5 degree steps to 50 or 0 °C, and then back to 25 °C. The resistance readings for the cycle of 25-0-25 °C were reproducible to within ±0.005%, while those for the cycle of 25-50-25 °C were not as good. The measured resistances for the reverse cycle from 50-25 °C were generally lower than the forward cycle (25 to 50 °C) due to evaporative loss of water from the cell. Hence, only those results for the forward cycle were used in the final analysis. In addition, at high temperatures (45 and 50 °C), small air bubbles would sometimes form in the chamber between electrodes, in which case higher resistance readings resulted. There was no easy way to get rid of the bubbles except to take the cell out and to rotate it. This process usually resulted in loss of water vapor, ending in lower resistance readings. It was preferable to rotate the cell while it was still in the bath, but this was only successful a third of the time. In most cases, the measurement had to be repeated when bubbles developed.

## 14. Results

All the values listed in the following tables were based on the measurements at the 1968 temperature scale (IPTS-68) from 0 to 50 °C in 5 degree increments. Currently, a new 1990 international temperature scale has been adopted (ITS-90) [6]. The portion of the new scale that affects our values (0 to 50 °C) can be described as

$$t_{90}-t_{68}=-2.6\times 10^{-4}t.$$ (8)

The corrected values for the electrolytic conductivities are listed separately.

The observed resistances, corrected for lead resistance, are listed in table 1. Calculated cell constants, based on the development outlined in section 2, are listed in table 2. The electrolytic conductivities, κ, of 0.01 m and 0.1 m KCl solutions, and of solvent are listed in table 3.

The temperature effect can be expressed as

$$\kappa_{68}=a+bt+ct^{2}+dt^{3}$$ (9)

where *a, b, c*, and *d* are temperature coefficient constants for κ. Their values for 0.01 *m* and 0.1 m KCl solutions are listed in table 4.

The change of κ_68_ with respect to *t* is

$$\frac{d\kappa_{68}}{dt}=b+2ct+3dt^{2}$$ (10)

Thus, the κ_90_ of the ITS-90 temperature scale will be

$$\kappa_{90}=\kappa_{68}+\left(b+2ct+3dt^{2}\right)\times 2.6\times 10^{-4}t.$$ (11)

The values for κ_90_ are listed in table 5.

## 5. Discussion

Accuracy is the necessary requirement for a primary standard. There are four main factors that could affect the accuracy of these results, viz., 1) the purity, stability, and accuracy of the concentration of the KCI solutions during preparation, storage, and measurement; 2) the cell constants and the cleanliness of the cell; 3) the temperature control; and 4) the measuring instrumentation. Of these, the last one caused the least problem (< 0.002%). With regard to the bath temperature, the stability and control attained were ±0.001 to ± 0.002 °C, except at 50, 5, and 0 °C, where the stability degraded to ±0.003, ±0.003, and ± 0.005 °C, respectively. The cell constants, *G*_w_ and *G*_N_, were permanently changed during the initial thermal cycling from 0 to 50 °C. After this, they were stable and consistent. Most likely, this was due to the graded glass used for sealing the platinum electrodes and the joint to the Pyrex glass. It is advised that any cell used for conductivity measurement over a wide range of temperature should be subjected to the same thermal cycle before it is calibrated. Thereafter, its stability should be monitored. The uncertainty in the values for electrolytic conductivity due to the measurement and maintenance of temperature is estimated not to exceed 0.01%.

Maintaining the cleanliness of the cell was not a simple problem. During the course of lowering the cell temperature, if the change was too abrupt, such as from room temperature to 0°C, the oil from the bath could be drawn into the cell. There was no easy way to remove the thin oil film adhering to the cell wall except to use chromic acid. However, the chromate ions tended to adsorb to the cell. It took about two weeks to leach out these ions by repeating soaking and refilling with high-purity water. The extent to which the cell is “clean” may be the controlling factor in the random uncertainty in the measurement (see below). The uncertainty in the cell constant is estimated to be 0.02%, as discussed in section 2.3.

The accuracy of the concentration of the KCl solutions, as limited by the purity, stability, and preparation, was estimated to be within ±0.005%. The least known effects resulted from evaporation, condensation and contamination during the course of transferring solution from the stock to the cell, and thermal cycling from one temperature to another. The solutions were occasionally contaminated (perhaps by the cell) as noted by the conductance drifting upward. These random events could compromise the reproducibility to ±0.01 to 0.02%,

Therefore, the total uncertainty in the electrolytic conductivity values over the entire range of temperatures was estimated conservatively to be approximately 0.03% (determined by the root sum square method of combining uncertainties).

There were at least three determinations made for each temperature and concentration. Each of these points consisted of two independent measurements, one with the center tube in place, and the other without the center tube. The difference in resistance between the two was used to determine the electrolytic conductivity, κ, as shown in eq (5).

To ensure that the κ’S obtained are consistent and accurate, eqs (2) and (3) have to be used to determine the same κ’S for verification.

In section 2.5, eq (7) shows that (*R*_w_/*R*_N_)_A_ = (*R*_w_*/R*_N_)_B_, which is the same as (*G*_W_/*G*_N_)_A_ = (*G*_W_/*G*_N_)_B_. It will also be true that

$$G_{w}/G_{T}=R_{w}/\Delta R,$$ (12)

$$G_{w}=\left(R_{w}/\Delta R\right)G_{T}$$ (13)

and

$$G_{N}=\left(R_{N}/\Delta R\right)G_{T}.$$ (14)

Since

$$G_{T}\left(t\right)=G_{T}\left(25\right)\;\left[1-\alpha_{g}\left(t-25\right)\right],$$ (15)

from the ratio of the observed resistances, i.e., the ratio of *G’s*, as a function of temperature the cell constants, *G*_w_ and *G*_N_ can be obtained by eqs (13), (14), and (15). Using eqs (2), (3), and (4), three sets of κ can be computed. They should be identical within experimental uncertainty.

It is possible, however, that both *R*_w_ and *R*_N_ may be off by some fraction, α, i.e., α*R*_w_/α*R*_N_ = *R*_w_/*R*_N_. In this case, either the κ or the *G*’s will be off by the same factor a. Statistically, we can compute the standard deviation from any one of the three elements, *R*, κ, and *G*, for they are interrelated. We selected *G*, because (a) it is a constant at a given temperature, (b) it is a linear function of temperature [eq (15)], (c) its change through the temperature range of 0–25 and 25 to 50 °C is small, and (d) at 25 °C we have accumulated more data to ensure its constancy.

**Figure 5:**
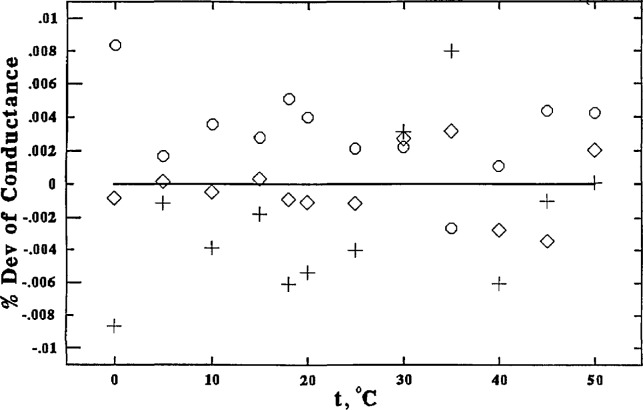
0.01 *m* KCl, percent deviations of values obtained using the three different cell configurations from the smoothed values (solid line): without center tube, ○; with center tube, ◊; center tube only, +.

**Figure 6:**
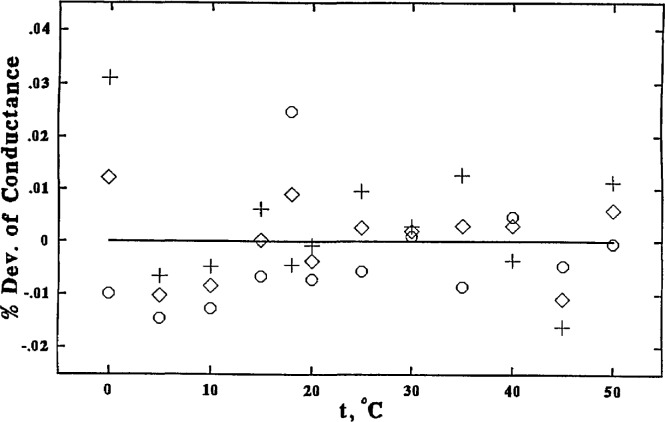
0.1 *m* KCl, percent deviations of values obtained using the three different cell configurations from the smoothed values (solid line): without center tube, ○; with center tube, ◊; center tube only, +.

Employing these smoothed cell constants, all the values for κ at all temperatures were recalculated and were fitted to a polynomial function of temperature with the method of least squares. The final values are shown in table 3. The differences between the observed and the smoothed values are plotted in figures 5 and 6.

Finally, a new temperature scale ITS-90 was adopted at the beginning of 1990 (after all the measurements had been done with IPTS-68 temperature scale). Therefore, the electrolytic conductivity values had to be adjusted to the new ITS-90 scale by eq (11). The adjusted values are shown in table 5. A few values at 25 and 40 °C were determined using a thermometer calibrated on the ITS-90 scale in order to validate the adjustment. These values were within 0.01% of the adjusted values. It is recommended that the electrolytic conductivity values listed in table 5 be adopted as the primary standards for electrolytic conductivity over the temperature range studied.

## Figures and Tables

**Table 1:** Observed resistance values for 0.01 *m* and 0.1 *m* KCl solutions (ohm)

*t*°C	*R*_w_	0.01 *m* *R*_N_	Δ*R*	*R*_w_	0.1 *m* *R*_N_	Δ*R*
0	24073.1	11067.20	13005.90	2615.85	1202.97	1412.88
5	20883.7	9601.68	11282.00	2275.40	1046.22	1229.18
10	18350.7	8436.89	9913.81	2004.06	921.46	1082.60
15	16301.0	7494.65	8806.35	1784.00	820.30	963.70
18	15252.6	7012.39	8240.21	1671.20	768.26	902.94
20	14616.6	6720.06	7896.54	1603.02	737.06	865.96
25	13213.3	6075.00	7138.30	1451.86	667.59	784.27
30	12030.5	5531.43	6499.11	1324.38	608.93	715.45
35	11024.4	5069.10	5955.30	1215.70	559.02	656.68
40	10161.3	4671.80	5489.50	1122.30	515.99	606.31
45	9414.10	4328.10	5086.00	1041.47	478.82	562.65
50	8762.80	4028.90	4733.90	970.70	446.34	524.36

**Table 2:** Cell constants, cm^−1^

*t*°C	*G*_w_	*G*_N_	*G*_T_
0	18.6204	8.56110	10.0594
5	18.6202	8.56105	10.0592
10	18.6200	8.56101	10.0590
15	18.6198	8.56096	10.0589
18	18.6197	8.56093	10.0588
20	18.6196	8.56091	10.0587
25	18.6194	8.56086	10.0585
30	18.6192	8.56082	10.0583
35	18.6190	8.56077	10.0581
40	18.6188	8.56072	10.0580
45	18.6185	8.56067	10.0578
50	18.6183	8.56062	10.0576

**Table 3:** Electrolytic conductivities for 0.01 *m* and 0.1 *m* KCI solutions, IPTS-68 scale (S/cm, H_2_O corrected)

*t*°C	0.01 *m* KCI	κ (S/cm) 0.1 *m* KCI	H_2_O
0	7.72921 × 10^−4^	7.11685 × 10^−3^	0.58 × 10^−6^
5	8.90932 × 10^−4^	8.18342 × 10^−3^	0.68 × 10^−6^
10	1.01389 × 10^−3^	9.29113 × 10^−3^	0.79 × 10^−6^
15	1.14135 × 10^−3^	1.04362 × 10^−2^	0.89 × 10^−6^
18	1.21981 × 10^−3^	1.11395 × 10^−2^	0.95 × 10^−6^
20	1.27289 × 10^−3^	1.16147 × 10^−2^	0.99 × 10^−6^
25	1.40805 × 10^−3^	1.28230 × 10^−2^	1.10 × 10^−6^
30	1.54641 × 10^−3^	1.40573 × 10^−2^	1.20 × 10^−6^
35	1.68753 × 10^−3^	1.53137 × 10^−2^	1.30 × 10^−6^
40	1.83097 × 10^−3^	1.65883 × 10^−2^	1.40× 10^−6^
45	1.97628 × 10^−3^	1.78776 × 10^−2^	1.51 × 10^−6^
50	2.12305 × 10^−3^	1.91775 × 10^−2^	1.61× 10^−6^

**Table 4:** Parameters for κ of eq (9) for 0.01 *m* and 0.1 *m* KCI solutions

	0.01 *m*	0.1 *m*
a	7.72921 × 10^−4^	7.11685 × 10^−3^
b	2.30786 × 10^−5^	2.08948 × 10^−4^
c	1.07659 × 10^−7^	8.98677 × 10^−7^
d	−5.83639 × 10^−10^	−5.06729× 10^−9^

**Table 5:** Electrolytic conductivities for 0.01 *m* and 0.1 *m* KCI solutions, recommended values ITS-90 scale

*t*°C	μS/cm	
	0.01 *m*	0.1 *m*
0	772.921	7116.85
5	890.961	8183.70
10	1013.95	9291.72
15	1141.45	10437.1
18	1219.93	11140.6
20	1273.03	11615.9
25	1408.23	12824.6
30	1546.63	14059.2
35	1687.79	15316.0
40	1831.27	16591.0
45	1976.62	17880.6
50	2123.43	19180.9

## Footnotes

1 Certain commercial equipment, instruments, or materials are identified in this paper to specify adequately the experimental procedure. Such identification does not imply recommendation or endorsement by the National Institute of Standards and Technology, nor does it imply that the materials or equipment identified are necessarily the best available for the purpose.
